# Effect of somatic maturity on the aerobic and anaerobic adaptations to sprint interval training

**DOI:** 10.14814/phy2.14426

**Published:** 2020-05-06

**Authors:** Kyle S. Beyer, Jeffrey R. Stout, Michael J. Redd, Kayla M. Baker, David D. Church, Haley C. Bergstrom, Jay R. Hoffman, David H. Fukuda

**Affiliations:** ^1^ Department of Exercise Science Bloomsburg University of Pennsylvania Bloomsburg PA USA; ^2^ Institute of Exercise Physiology and Rehabilitation Science University of Central Florida Orlando FL USA; ^3^ Center for Translation Research in Aging & Longevity University of Arkansas for Medical Sciences Little Rock AR USA; ^4^ Department of Kinesiology and Health Promotion University of Kentucky Lexington KY USA; ^5^ Department of Molecular Biology Ariel University Ariel Israel

**Keywords:** fatigue thresholds, maturation, years from peak height velocity, youth athletes

## Abstract

The purpose of this study was to assess the maturity‐related differences in the aerobic and anaerobic adaptations to sprint interval training (SIT) among youth male athletes. Twenty‐seven youth male athletes were assessed for years from peak height velocity (PHV) and classified into prepubescent (PRE, *n* = 7, years from PHV = −2.21 ± 0.47 years), peripubescent (PERI, *n* = 10, years from PHV = 0.25 ± 0.88 years), and postpubescent (POST, *n* = 10, years from PHV = 2.81 ± 0.50 years) groups based on their years from estimated peak height velocity. Participants completed a ramp exercise protocol on a cycle ergometer to determine maximal aerobic power, maximal oxygen consumption (VO_2peak_), and fatigue thresholds. Following baseline, all participants completed a 4‐week SIT program that consisted of eight total training sessions. During each session, participants completed repeated 20‐s sprints on a cycle ergometer against a resistance of 7.5% of body mass. The number of sprints per sessions increased from four in session 1 to seven in session 7, with four sprints in session 8. Peak and mean power from sessions 1 and 8 were recorded. All participants completed a post‐testing ramp exercise protocol that mirrored baseline. Maximal aerobic power increased (*p* < .001) across all groups from baseline (212.61 ± 57.45 W) to post‐testing (223.24 ± 58.90 W); however, VO_2peak_ only increased in POST (3.31 ± 0.43 to 3.54 ± 0.43 L min^−1^, *p* = .003). Similarly, GET, VT, and RCP increased in POST, with no changes in PRE or PERI. In terms of anaerobic performance, PERI and POST had significant increases in peak and mean power. POST improved aerobic and anaerobic performance following SIT, while PERI only experienced improvements in anaerobic performance. Conversely, PRE had no changes in aerobic or anaerobic performance. The adaptations to SIT appear to be influenced by the somatic maturity status.

## Abstract 2

After 4 weeks of sprint interval training, improvements in aerobic and anaerobic exercise performance were observed in youth male athletes. However, prepubertal participants had no changes following training.

## 1 INTRODUCTION

Prior to peak height velocity (PHV), prepubescent (PRE) children tend to have a reduced anaerobic capacity and a greater reliance on aerobic metabolism during exercise when compared to adults (Boisseau & Delamarche, 2000). The metabolic response to exercise in PRE children is characterized by lower lactate production following high‐intensity exercise (Beneke, Hütler, & Leithäuser, 2007) and greater fat oxidation rates than postpubescent (POST) and young adult males (Riddell, 2008; Rowland & Boyajian, 1995). Furthermore, POST youth have similar metabolic responses to exercise as young adult males, indicating a maturity‐related change in the metabolic response to exercise. Previous research has suggested that PRE youth rely more on aerobic metabolism during submaximal exercise than POST youth due to their underdeveloped anaerobic system (Boisseau & Delamarche, 2000). These maturity‐related differences in the metabolic response to exercise may be observed in anaerobic, or fatigue, thresholds, which have been previously demonstrated to occur at lower absolute intensities in children when compared to adults (Anderson & Mahon, 2007; Klentrou, Nishio, Plyley, & University, 2006; Pitt et al., 2015). Furthermore, maturity status has been shown to influence the onset of systemic pulmonary and localized muscular fatigue thresholds, with fatigue thresholds occurring at higher relative intensities in PRE when compared to POST (Beyer et al., 2019). While the trainability of many fatigue thresholds has been observed in youth (Barker, Day, Smith, Bond, & Williams, 2014; Faude, Schnittker, Schulte‐Zurhausen, Müller, & Meyer, 2013; Mucci et al., 2013; Rotstein, Dotan, Bar‐Or, & Tenenbaum, 1986), a direct comparison of the training adaptations to fatigue thresholds across maturation groups has not been investigated.

In terms of anaerobic exercise performance, previous literature has concluded that muscular power output is related to age and maturation (Lloyd & Oliver, 2012; Williams, 1997). Ratel, Williams, Oliver, & Armstrong (2004) observed less absolute and relative peak power (PP) and mean power (MP) during repeated sprints in PRE youth when compared to adult men. However, the relative declines in PP and MP during the repeated sprints were greater in adult men when compared to PRE, possibly due to the PRE boys having a blunted blood lactate accumulation response to the repeated sprints (Ratel et al., 2004). Similar to the findings in adults, Bottaro and colleagues (2011) observed reduced torque output and a blunted increase in blood lactate concentrations in PRE when compared to POST during repeated isokinetic contractions. Furthermore, PRE have been shown to have lower relative PP and MP during a single Wingate test when compared to POST (Beneke et al., 2007), but to the best of our knowledge no study has assessed how training and maturity status impact repeated anaerobic exercise performance.

Previous studies suggest that maturation status may have an effect on training‐induced adaptations (Behringer, Heede, Yue, & Mester, 2010; Lesinski, Prieske, & Granacher, 2016; Lloyd, Radnor, Croix, Cronin, & Oliver, 2016). For example, Behringer et al. (2010) demonstrated greater strength and power adaptations to resistance training in peripubescent (PERI) and POST children when compared to PRE. However, a recent meta‐analysis reported no significant effect of maturity on strength, power, sprint, or agility adaptations to resistance training (Lesinski et al., 2016). Moreover, a plyometric‐only training program resulted in greater jumping and sprinting adaptations in PRE children when compared to POST children (Lloyd et al., 2016). While maturity‐related differences in response to resistance and plyometric training have been thoroughly researched, limited research has directly compared the effect of maturity status on the adaptations to high‐intensity interval training (HIIT) or sprint interval training (SIT). Sprint interval training (SIT) focuses on supramaximal exercise intensities for very short (~10–30 s) durations of time. Previous research has shown that recreationally active adults engaging in SIT or HIIT can improve ventilatory threshold and respiratory compensation point (McKay, Paterson, & Kowalchuk, 2009), increase physical working capacity at fatigue threshold in (Riffe et al., 2017), and oxygen uptake by exercising muscle (McKay et al., 2009).

In adolescents, SIT has been shown to improve peak power, gas exchange threshold, muscle thickness, fatigue index, $\dot{V}$O_2_max, and time to exhaustion after eight training sessions over 2 weeks (Barker, Day, et al., 2014). In terms of anaerobic exercise performance, Barker, Day, et al. (2014) observed significant increases in repeated PP, but not MP, following 2 weeks of SIT in POST. Only one study has directly compared the maturity‐related differences to HIIT and observed improvements in agility and intermittent running performance among POST girls, while the PRE and PERI had smaller improvements, and some performance decrements (Wright, Hurst, & Taylor, 2016). Boys typically experience a greater adolescent growth spurt than girls and may experience greater maturity‐related differences in the adaptation to training compared to girls (Beunen & Malina, 1988). Currently, no study has directly compared the aerobic and anaerobic adaptations to SIT between youth boys at different stages of maturation. Maturational status may have an impact on adaptations to training, due to differences in the utilization and adaptability of aerobic and anaerobic energy systems and the maturing neuromuscular system.

The purpose of this study was to compare aerobic and anaerobic adaptations following SIT in youth males of different maturational status. Based on the results of previous studies, we hypothesized that POST children would see increases in maximal aerobic performance, fatigue thresholds, and repeated anaerobic performance following 4 weeks of SIT. In terms of the PERI children, it was expected that SIT would improve in all variables, but to a lesser extent than the POST group. Finally, we expected limited adaptations in PRE, particularly with regards to repeated anaerobic performance as they have an underdeveloped anaerobic energy system.

## 2 METHODS

### 2.1 Participants

Thirty‐three youth male athletes were recruited for this study. All participants were between the ages of 11 and 17 years old, and were actively engaged in a competitive sport and maintained habitual activity throughout the study. Participants were considered multisport athletes, competing in at least two different sports during the previous year. With the help of a parent, each participant completed a Confidential Medical and Activity Questionnaire, and provided a cleared physical to play sports from a medical doctor within the last year. Outside sport practices were maintained throughout the study, but participants were not allowed to use any ergogenic nutritional supplements. The parents of all participants provided informed consent prior to beginning the study, along with a verbal assent from the participant. Six participants did not complete the study due to scheduling conflicts. This study was approved by the University Institutional Review Board and was conducted in accordance with the Declaration of Helsinki.

### 2.2 Research design

This study utilized a quasi‐experimental design to compare the effects of a 4‐week SIT program on maximal aerobic performance, systemic pulmonary and localized muscular fatigue thresholds, and repeated anaerobic sprint performance in youth males of different maturational groups. Participants were grouped according to their number of years from peak height velocity (PHV), an estimation of somatic maturity status, into PRE‐, PERI‐, and POST‐PHV groups. All participants completed a single pretesting session (T1), eight SIT sessions, and a single post‐testing session (T2). Measures of age, years from PHV, height, and body mass for each group assessed at T1 are presented in Table 1.

**TABLE 1:** Pretesting (T1) descriptive data

	All (*n* = 27)	PRE (*n* = 7)	PERI (*n* = 10)	POST (*n* = 10)
Chronological age (years)	14.63 ± 2.33	11.57 ± 0.57	14.33 ± 1.12	17.06 ± 0.60
Years from PHV (years)	0.56 ± 2.11	−2.21 ± 0.47	0.25 ± 0.88	2.81 ± 0.50
Height (cm)	166.63 ± 14.10	148.37 ± 8.81	167.56 ± 6.92	178.48 ± 7.64
Mass (kg)	59.13 ± 17.59	39.8 ± 7.05	55.07 ± 6.71	76.76 ± 12.75
Body fat (%)	15.68 ± 8.11	20.89 ± 11.43	14.45 ± 4.68	13.27 ± 7.21

Note

PHV, peak height velocity.

### 2.3 Testing sessions

Each testing session consisted of PHV estimation and a ramp exercise protocol. Participants reported to the testing session in a euhydrated state and fasted for a period of 4 hr. All testing sessions were completed at a similar time of day, and occurred at least 48 hr before or after the SIT program.

#### 2.3.1 Peak height velocity estimation methods

Each participant's years from PHV was estimated using standing height, seated height, leg length, body mass, and age using methods from Mirwald, Baxter‐Jones, Bailey, & Beunen (2002). Standing height and body mass were measured using a digital scale (Model 500 Kl, Health‐o‐meter Professional Scales, McCook, IL, USA). Seated height was measured with the participant seated on a bench from the base of the seat to the top of the head using a tape measure. Leg length was calculated by subtracting seated height from standing height. Equation 1 was used to calculate years from PHV (Mirwald et al., 2002). This equation has been previously established to be accurate within 1 year 95% of the time (*R* ^2^ = .891, *SE* of estimate = 0.59 years) (Mirwald et al., 2002).

(1) $$\begin{array}{cc} \text{Years from PHV} & =-9.236+\left(0.0002708\times \left(\text{Leg length}\times \text{Seated height}\right)\right)\\ & \;+\left(-0.001663\times \left(\text{Age}\times \text{Leg length}\right)\right)\\ & \;+\left(0.007216\times \left(\text{Age}\times \text{Seated}\;\text{height}\right)\right)\\ & \;+\left(0.02292\times \left(\left(\frac{\text{Weight}}{\text{Height}}\right)\times 100\right)\right) \end{array}$$

**FIGURE 1:**
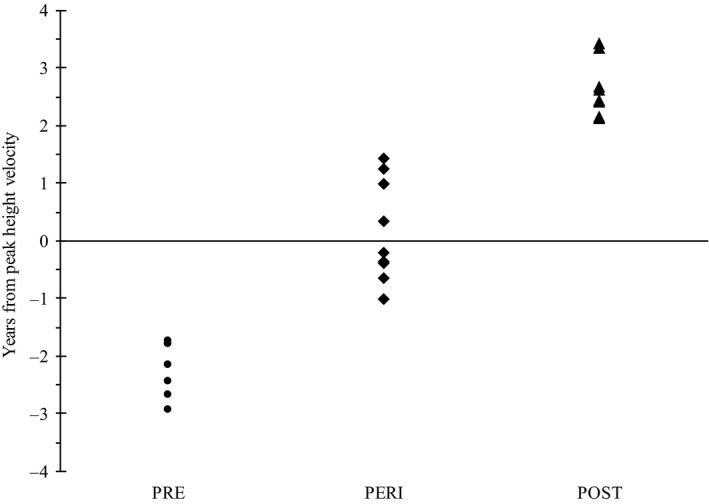
Years from Peak Height Velocity for each participant within prepubescent (PRE), peripubescent (PERI), and postpubescent (POST) maturity groups

The cutoff for each maturity group was less than −1.5 (PRE, *n* = 7), greater than + 1.5 (POST, *n* = 10), and between −1.5 and + 1.5 (PERI, *n* = 10). The years from PHV for each subject, separated by maturity group, are presented in Figure 1.

#### 2.3.2 Ramp exercise protocol methods

The ramp exercise protocol and fatigue threshold determination methods were adapted from Beyer et al. (2019). Briefly, the ramp exercise protocol required each participant to be equipped with electromyography (EMG) and near‐infrared spectroscopy (NIRS) on each leg, while expired breaths were captured and analyzed using a metabolic cart. After equipment setup, each participant was seated on a cycle ergometer (Lode, Excalibur Sport), and the handle and seat heights were adjusted to a comfortable position and replicated for both T1 and T2. Following a 3‐min rest period to allow for acclimatization to the equipment and normalize all physiological readings, there was a 3‐min warm‐up of cycling at a workload of 0 watts. Immediately after the warm‐up, the ramp protocol began with an initial workload of 30 watts, which increased 1 watt every 3 s (20 watts min^−1^). Throughout the entire test, each participant was required to maintain 65–85 revolutions per minute. The test was terminated when the participant could not maintain 65 revolutions per minute despite strong verbal encouragement. The workload that each participant achieved at volitional fatigue was recorded as maximal aerobic power.

#### 2.3.3 Metabolic methods

To assess oxygen consumption ($\dot{V}$O_2_), carbon dioxide production ($\dot{V}$CO_2_), and ventilation ($\dot{V}$E) during the ramp exercise protocol, a flexible mask was fitted over each participant's mouth and nose to collect expired air. After setup, each mask was tested, via a forcible exhalation, to ensure that no air escaped while breathing. The expired air was sampled and analyzed by a metabolic cart (TrueOne 2400, Parvo Medics). $\dot{V}$O_2_, $\dot{V}$CO_2_, and $\dot{V}$E were measured and 10‐s averages were calculated. Peak oxygen consumption ($\dot{V}$O_2_peak) was determined as the highest recorded 30‐s average when the respiratory exchange ratio was greater than 1.15 and heart rate was greater than 85% of age‐predicted maximum.

#### 2.3.4 Electromyography methods

To assess muscle activity during the ramp exercise protocol, a bipolar (4.6 cm center‐to‐center) surface electrode (Quinton Quick‐Prep silver‐silver chloride) arrangement was placed over the vastus lateralis muscle of each leg, at approximately 66 percent of the line from the anterior superior illiac spine to the superior lateral border of the patella (Beyer et al., 2019). The reference electrode was placed over Gerdy's tubercle. Interelectrode impedance was kept below 5,000 ohms with shaving and abrasion of the skin beneath the electrodes. The raw EMG signals were preamplified using a differential amplifier (BioNomadix 2‐Channel EMG, BIOPAC Systems, Inc.), sampled at 1,000 Hz, and stored on a personal computer (Dell Latitude E6530, Dell Inc.) for offline analysis.

Using computer software (AcqKnowledge v4.2, BIOPAC Systems, Inc.), the raw EMG data were filtered using a bandpass Butterworth filter at 10–500 Hz. From the filtered signals, root mean square (RMS) was calculated of the vastus lateralis from each leg. Averages were calculated for each 10‐s epoch of the ramp exercise protocol.

#### 2.3.5 Near‐infrared spectroscopy methods

To assess tissue oxygenation during the ramp exercise protocol, a NIRS optode (PortaLite, Artinis Medical Systems) was placed over the vastus lateralis muscle of each leg, lateral to the previously stated EMG placement. The optode was secured using a self‐adhering bandage. A modified form of the Beer‐Lambert Law was used to calculate micromolar changes in oxygenated hemoglobin (O_2_Hb) and deoxygenated hemoglobin (HHb) during the ramp exercise protocol. Tissue oxygenation was then calculated by subtracting HHb from O_2_Hb to determine the balance between oxygen supply and oxygen consumption. Averages for each measure were calculated for each 10‐s epoch of the ramp exercise protocol.

#### 2.3.6 Threshold determination methods

**FIGURE 2:**
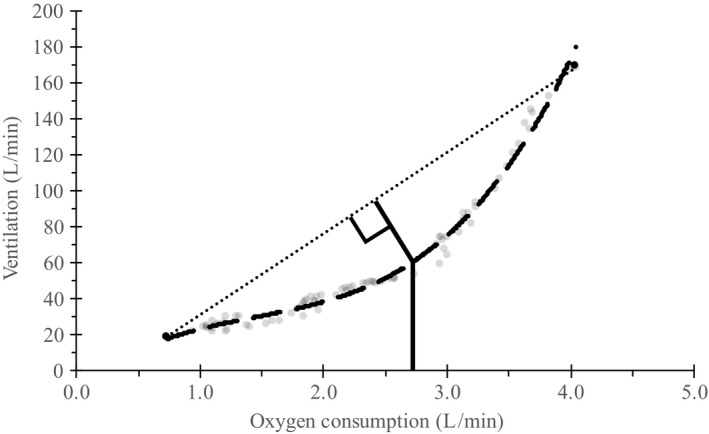
Example of the maximal deviation method used to determine fatigue thresholds. Actual participant data are presented to determine ventilatory threshold

All fatigue thresholds were determined using methodologies previously outlined by Beyer et al. (2019). The three systemic pulmonary fatigue thresholds that were determined were gas exchange threshold (GET), ventilatory threshold (VT), and respiratory compensation point (RCP). GET was determined as the nonlinear inflection in the relationship between $\dot{V}$CO_2_ and $\dot{V}$O_2_. VT was determined as the nonlinear inflection in the $\dot{V}$E versus $\dot{V}$O_2_ relationship. RCP was the $\dot{V}$O_2_ value corresponding to the nonlinear inflection in the $\dot{V}$E versus $\dot{V}$CO_2_ relationship. The three localized muscular thresholds were neuromuscular fatigue threshold (NFT), deoxyhemoglobin breakpoint (HHbBP), and oxygenation deflection point (OxDP). NFT was determined from the nonlinear inflection in the RMS, from the vastus lateralis versus $\dot{V}$O_2_ relationship. HHbBP was determined as the nonlinear deflection in the HHbBP versus $\dot{V}$O_2_ relationship. OxDP was determined as the nonlinear deflection in the tissue oxygenation versus $\dot{V}$O_2_ relationship. Localized muscular fatigue thresholds were determined for each leg and averaged between the two legs. All fatigue thresholds were determined using the maximal deviation (*D*_max_) methodology. For each physiological variable, 30‐s moving averages were calculated from the 10‐s averages obtained from each respective software. The 30‐s moving averages were then plotted on a graph versus $\dot{V}$O_2_, and the data points were fitted with a third‐order polynomial regression line. Then, a linear regression line was computed from the first and last data points. The point on the third‐order polynomial line of best fit that was the furthest perpendicular distance from the linear line was considered the fatigue threshold. Equation 2 was utilized to calculate the *D*_max_ point (Machado, Nakamura, & Moraes, 2012).

(2) $$D_{\max}=\frac{\left\{-b\pm \sqrt{\left[\left(b^{2}-3\times a\left(c-\Delta \right)\right)\right]}\right\}}{3\times a}$$

where *a*, *b*, and *c* are the parameters of the third‐order polynomial equation and delta (∆) is the slope of the linear line connecting the first and last data points. An example of how the *D*_max_ method was used to determine ventilatory threshold is presented in Figure 2. Previously we examined the test–retest reliability for all threshold measures in young male athletes (*n* = 29; age 14.62 ± 2.39 y) and reported ranges for ICC of 0.911 to 0.81 and *SEM* of 0.078 L min^−1^ to 0.146 L min^−1^ (Beyer et al., 2019).

### 2.4 Sprint interval training

After completing T1, participants completed a 4‐week sprint interval training program. The training program was adapted from previously published research (Barker, Day, et al., 2014). This training program was chosen as it has been previously shown to be beneficial for maximal aerobic, submaximal aerobic, and anaerobic exercise performance in POST (Barker, Day, et al., 2014). Training sessions were conducted 2 days per week, with at least 24 hr of rest between each training session. Each training session consisted of 20‐s maximal sprints on a cycle ergometer (894E, Monark Exercise AB) against a load equivalent to 7.5% of the participant's body mass. Prior to beginning each training session, participants completed a 5‐min warm‐up at a self‐selected intensity, with intermittent practice sprints. Participants were instructed to perform each sprint “as fast as possible”. Prior to each sprint, participants were provided a 3‐s countdown, during which time they were instructed to rapidly increase their RPM. Once participants achieved 120 RPM, the sprint load was applied to the cycle ergometer. A 4‐min active recovery period was provided between each sprint. The sprint interval training program was progressive, with an additional sprint added each week, except for the last training session which served as a taper. During the first (SIT1) and last (SIT8) sessions PP and MP were recorded for each of the four sprints. Additionally, total work (TW) was calculated for each sprint to determine training volume. The training program is presented in Table 2.

**TABLE 2:** Sprint interval training program

Training session	Number of sprints	Resistance	Duration of sprints	Duration of recovery	Total session time
Week 1	4	7.5% of body mass	20 s	4 min	14 min
Day 1			(1.3 min total)		
Week 1	4	7.5% of body mass	20 s	4 min	14 min
Day 2			(1.3 min total)		
Week 2	5	7.5% of body mass	20 s	4 min	18.5 min
Day 1			(1.7 min total)		
Week 2	5	7.5% of body mass	20 s	4 min	18.5 min
Day 2			(1.7 min total)		
Week 3	6	7.5% of body mass	20 s	4 min	23 min
Day 1			(2.0 min total)		
Week 3	6	7.5% of body mass	20 s	4 min	23 min
Day 2			(2.0 min total)		
Week 4	7	7.5% of body mass	20 s	4 min	27.5 min
Day 1			(2.3 min total)		
Week 4	4	7.5% of body mass	20 s	4 min	14 min
Day 2			(1.3 min total)		

### 2.5 Statistical analysis

All data are reported as mean ± *SD*. Changes from T1 to T2 for all maximal aerobic performance and fatigue threshold data, and the changes in session average PP and MP from SIT1 to SIT8 data were calculated. Maximal aerobic performance data and each fatigue threshold were analyzed with separate two‐way [group (PRE vs. PERI vs. POST) × time (T1 vs. T2)] mixed factorial analysis of variance (ANOVA). Any statistically significant group × time interactions were followed by Bonferroni‐adjusted dependent *t* tests for each group and one‐way ANOVAs at T1, T2, and for changes from T1 to T2. Changes in PP and MP from SIT1 to SIT8 were assessed using three‐way [group (PRE vs. PERI vs. POST) × sprint (1 vs. 2 vs. 3 vs. 4) × time (SIT1 vs. SIT8)] mixed factorial ANOVAs. Statistically significant group × time interactions were followed with a Bonferroni‐adjusted dependent *t* test for each group and a one‐way ANOVA at SIT1, SIT8, and for changes with data averaged across sprints. One‐way ANOVA was conducted to assess group differences in TW during training. All main effects of group and time were followed with Bonferroni‐adjusted pairwise comparisons averaged across time and group, respectively. Statistical software (SPSS; V25.0; SPSS, Inc) was used for all parametric statistics. Results were considered statistically significant at an alpha level of *p* ≤ .05. Additionally, Cohen's d coefficients were calculated when comparing changes between groups and were interpreted as small (0.2), moderate (0.5), and large (0.8) (Cohen, 1988).

## 3 RESULTS

### 3.1 Maximal aerobic performance

Maximal aerobic performance data are presented in Table 3. No group × time interaction (*F* = 1.770, *p* = .192) was observed for maximal aerobic power; however, a main effect of time (*F* = 17.62, *p* < .001) was observed as an increase in maximal aerobic power from T1 to T2 regardless of group. For $\dot{V}$O_2_peak, a group × time interaction (*F* = 3.948, *p* = .033) was observed, with statistical differences noted between all three groups at both T1 and T2 (*p* < .002). No significant changes in $\dot{V}$O_2_peak were seen for either PRE (*p* = .410) or PERI (*p* = .501) from T1 to T2; however, POST experienced a significant (*p* = .003) increase in $\dot{V}$O_2_peak from T1 (3.31 ± 0.43 L min^−1^) to T2 (3.54 ± 0.43 L min^−1^). When comparing the changes in $\dot{V}$O_2_peak between groups, no significant difference was observed between PRE and PERI (*p* ≥ .999); *d* = 0.075). Furthermore, the changes in $\dot{V}$O_2_peak for POST were not significantly different than the changes in PRE (*p* = .076) or PERI (*p* = .065); however, Cohen's d coefficients revealed large differences when comparing the changes in $\dot{V}$O_2_peak between POST and PRE (*d* = 1.353) and POST and PERI (*d* = 0.982).

**TABLE 3:** Maximal aerobic power and peak oxygen consumption (VO_2peak_) at pretesting (T1), post‐testing (T2), and the change from T1 to T2 (Δ) in prepubescent (PRE), peripubescent (PERI), postpubescent (POST), and for the combined sample (ALL). Data presented as mean ± *SD*

Group	Maximal aerobic power (W)			$\dot{V}$O_2peak_ (L min^−1^)		
	T1	T2	Δ	T1	T2	Δ
PRE	136.00 ± 30.46	140.91 ± 23.18	4.92 ± 13.24	1.62 ± 0.31	1.65 ± 0.28	0.03 ± 0.09
PERI	217.09 ± 26.01	226.36 ± 18.62	9.27 ± 14.04	2.64 ± 0.29	2.69 ± 0.24	0.04 ± 0.20
POST	261.76 ± 31.13	277.75 ± 27.11	15.99 ± 9.40	3.31 ± 0.43	3.54 ± 0.43*	0.23 ± 0.19
ALL	212.61 ± 57.45	223.24 ± 58.90*	10.63 ± 12.64	2.62 ± 0.75	2.73 ± 0.82	0.11 ± 0.19

* Denotes significantly greater than T1.

### 3.2 Fatigue thresholds

Fatigue threshold data are presented in Tables 4 and 5. When assessing systemic pulmonary fatigue thresholds, significant group × time interactions for GET (*F* = 4.326, *p* = .025), VT (*F* = 4.345, *p* = .025), and RCP (*F* = 3.439, *p* = .049) were observed. At T1, significant group differences were observed for GET when comparing PRE versus PERI (*p* = .001) and PRE versus POST (*p* < .001); furthermore, VT and RCP had significant differences between all three groups at T1 (*p* < .030). Similar differences were observed between groups for GET, VT, RCP at T2, with the added difference in PERI versus POST for GET (*p* = .002). When assessing the changes from T1 to T2 in each group, POST had significant increases in GET (*p* = .009), VT (*p* = .009), and RCP (*p* = .007), but PRE and PERI experienced no significant changes in GET, VT, or RCP. Furthermore, when comparing changes in GET, VT, and RCP between groups, a significant main effect of group was noted for GET (*p* = .028) and VT (*p* = .021), with a trend for RCP (*p* = .052). Post hoc pairwise comparisons revealed significantly greater changes in POST when compared to PERI for GET (*p* = .044; *d* = 1.081) and VT (*p* = .031; *d* = 1.105). While the differences between PRE and POST for changes in GET (*p* = .102) and VT (*p* = .090) were not significant, both differences were considered large when interpreting Cohen's d (*d* = 1.364 and *d* = 1.417, respectively).

**TABLE 4:** Gas exchange threshold (GET), ventilatory threshold (VT), and respiratory compensation point (RCP) at pretesting (T1), post‐testing (T2), and the change from T1 to T2 (Δ) in prepubescent (PRE), peripubescent (PERI), postpubescent (POST), and for the combined sample (ALL). Data presented as mean ± *SD*

Group	GET (L min^−1^)			VT (L min^−1^)			RCP (L min^−1^)		
	T1	T2	Δ	T1	T2	Δ	T1	T2	Δ
PRE	1.12 ± 0.21	1.09 ± 0.18	−0.02 ± 0.10	1.23 ± 0.25	1.21 ± 0.22	−0.01 ± 0.08	1.37 ± 0.27	1.36 ± 0.24	−0.01 ± 0.11
PERI	1.72 ± 0.26^†^	1.69 ± 0.23^†^	−0.03 ± 0.20	1.92 ± 0.25^†^	1.89 ± 0.21^†^	−0.03 ± 0.17	2.10 ± 0.27^†^	2.07 ± 0.18^†^	−0.03 ± 0.19
POST	1.96 ± 0.33^†^	2.12 ± 0.31*†#	0.16 ± 0.15^#^	2.31 ± 0.39^†#^	2.44 ± 0.29*†#	0.14 ± 0.13^#^	2.54 ± 0.38^†#^	2.68 ± 0.31*†#	0.13 ± 0.12
ALL	1.65 ± 0.43	1.69 ± 0.48	0.05 ± 0.18	1.88 ± 0.52	1.92 ± 0.54	0.04 ± 0.15	2.07 ± 0.56	2.11 ± 0.58	0.04 ± 0.16

* Denotes a significant increase from T1.

^†^ Denotes significantly greater than PRE.

^#^ Denotes significantly greater than PERI.

**TABLE 5:** Neuromuscular fatigue threshold (NFT), oxygenation deflection point (OxDP), and deoxyhemoglobin breakpoint (HHbBP) at pretesting (T1), post‐testing (T2), and the change from T1 to T2 (Δ) in prepubescent (PRE), peripubescent (PERI), postpubescent (POST), and for the combined sample (ALL). Data presented as mean ± *SD*

Group	NFT (L min^−1^)			OxDP (L min^−1^)			HHbBP (L min^−1^)		
	T1	T2	Δ	T1	T2	Δ	T1	T2	Δ
PRE	1.34 ± 0.27	1.45 ± 0.30	0.11 ± 0.08	1.11 ± 0.18	1.15 ± 0.20	0.05 ± 0.10	1.38 ± 0.28	1.52 ± 0.25	0.15 ± 0.08
PERI	2.13 ± 0.26	2.22 ± 0.23	0.09 ± 0.09	1.51 ± 0.24	1.48 ± 0.27	−0.03 ± 0.25	1.98 ± 0.37	2.14 ± 0.32	0.16 ± 0.35
POST	2.57 ± 0.33	2.74 ± 0.38	0.18 ± 0.21	1.97 ± 0.49	2.14 ± 0.50	0.17 ± 0.25	2.45 ± 0.8	2.90 ± 0.37	0.45 ± 0.44
ALL	2.09 ± 0.56	2.21 ± 0.59*	0.13 ± 0.15	1.58 ± 0.48	1.64 ± 0.54	0.06 ± 0.23	2.00 ± 0.53	2.26 ± 0.64*	0.26 ± 0.36

* Denotes significant increase from T1.

For localized muscular fatigue thresholds, no significant group × time interactions were noted for NFT (*F* = 1.017, *p* = .377), HHbBP (*F* = 2.283, *p* = .124), or OxDP (*F* = 2.111, *p* = .143). However, significant main effects of time were observed for NFT (*F* = 18.793, *p* < .001) and HHbBP (*F* = 13.743, *p* = .001), but not for OxDP (*F* = 1.809, *p* = .191). Post hoc pairwise comparisons revealed significant increases in NFT and HHBbBP when averaged across maturity groups.

### 3.3 Repeated anaerobic performance

PP and MP from SIT1 and SIT8 are presented in Table 6. For PP, no group × sprint×time interaction was (*F* = .436, *p* = .853) observed; however, there was a group × time interaction (*F* = 3.883, *p* = .035). Post hoc tests revealed that session average PP was significantly different among all three groups at SIT1 and SIT8. Furthermore, PRE had no change (*p* = .169) in session average PP, while PERI (*p* = .003) and POST (*p* = .001) significantly increased from SIT1 to SIT8. However, the change in session average PP was similar between PERI and POST (*p* = 1.000; *d* = 0.145). A significant difference between PRE and PERI was noted for the change in session average PP (*p* = .044), which was determined to be a large difference (*d* = 1.355) favoring PERI. While the change in session average PP was not significantly different (*p* = .092) when comparing POST and PRE, Cohen's D coefficients revealed a large difference (*d* = 1.465) favoring POST.

**TABLE 6:** Peak power (PP) and mean power (MP) during the first (SIT1) and last (SIT8) sprint interval training sessions in prepubescent (PRE), peripubescent (PERI), postpubescent (POST), and for the combined sample (ALL). Data presented as mean ± *SD*

	Group	SIT 1				SIT 8			
		Sprint 1	Sprint 2	Sprint 3	Sprint 4	Sprint 1	Sprint 2	Sprint 3	Sprint 4
PP (W)	PRE	342.3 ± 73.4	363.4 ± 88.9	355.0 ± 75.9	367.5 ± 76.7	360.4 ± 67.9	387.8 ± 62.3	389.3 ± 75.0	382.1 ± 74.8
	PERI*#	658.8 ± 130.1	659.0 ± 128.2	629.2 ± 110.0	618.4 ± 76.8	762.6 ± 138.7	787.8 ± 142.5	753.4 ± 125.2	741.7 ± 125.7
	POST*	926.9 ± 281.9	972.1 ± 296.4	924.5 ± 272.8	845.0 ± 208.9	1,046.4 ± 270.8	1,079.1 ± 255.7	1,010.2 ± 246.1	964.2 ± 225.6
	ALL	676.1 ± 298.2	698.3 ± 312.3	667.5 ± 289.0	637.3 ± 234.2	763.4 ± 328.1	792.0 ± 326.0	754.1 ± 297.9	730.9 ± 279.5
MP (W)	PRE	268.8 ± 69.1	265.1 ± 65.8	257.5 ± 62.6	254.6 ± 49.3	257.5 ± 52.9	251.2 ± 59.4	245.8 ± 57.4	250.0 ± 58.0
	PERI*	488.2 ± 75.1	464.7 ± 57.0	438 ± 43.6	418.5 ± 41.3	509.4 ± 72.0	488.6 ± 62.8	464.5 ± 58.3	457.1 ± 52.3
	POST*#	684.3 ± 120.9	656.6 ± 132.0	604.1 ± 124.8	548.1 ± 82.6	729.4 ± 111.8	677.6 ± 97.6	637.4 ± 107.1	610.9 ± 99.9
	ALL	503.9 ± 188.7	484.0 ± 180.7	452.7 ± 161.6	424 ± 131.1	525.6 ± 205.4	497.1 ± 185.2	471.9 ± 173.9	460.4 ± 160.7

* Denotes session average for this group increased from SIT1 to SIT8.

^#^ Denotes the change in session average for this group was significantly greater than PRE.

Similar to PP, there was no group × sprint×time interaction (*F* = .606, *p* = .657) for MP; however, there was a group × time interaction (*F* = 4.524, *p* = .022). Post hoc tests revealed that session average MP was different among all three groups at SIT1 and SIT8. Furthermore, PRE had no change (*p* = .480) in session average MP, while PERI (*p* = .009) and POST (*p* = .012) significantly increased from SIT1 to SIT8. When comparing the changes in session average MP, there were no differences between PRE and PERI (*p* = .113) or PERI and POST (*p* ≥ .999). However, Cohen's *d* coefficients revealed that a large difference existed when comparing the changes in session average MP between PRE and PERI (*d* = 1.190) and a small difference existed between PERI and POST (*d* = 0.377). Furthermore, POST had a greater (*p* = .021) change in session average MP than PRE, which was determined to be a large difference (*d* = 1.313).

In terms of total work completed during training, absolute work was significantly different among all three groups (*p* < .001). For total work during training relative to body mass, a main effect of group was noted (*p* = .004); however, post hoc tests revealed no difference (*p* ≥ .999) between PERI and POST, while both PERI (*p* = .008) and POST (*p* = .009) completed more relative work than PRE.

## 4 DISCUSSION

The purpose of this study was to assess how maturity status affected the aerobic and anaerobic adaptations to a 4‐week sprint interval training program among youth male athletes. The primary finding of this study was that POST had significant improvements in all measures of maximal aerobic performance, average fatigue threshold, and repeated sprint performance. PERI experienced improvement in only repeated sprint performance, while PRE experienced no changes in any of the measured variables.

While many reviews and meta‐analyses have supported the trainability of $\dot{V}$O_2_max in response to high‐intensity interval training among children of all maturity groups (Costigan, Eather, Plotnikoff, Taaffe, & Lubans, 2015; Logan, Harris, Duncan, & Schofield, 2014), this study is unique in providing a direct comparison of the adaptations to the same sprint interval training program between maturity groups among youth males. In this study, a significant improvement in $\dot{V}$O_2_peak (0.23 ± 0.19 L min^−1^) was only observed in POST after 4 weeks of sprint interval training. Barker, Day, et al. (2014) observed significant increases in absolute and relative $\dot{V}$O_2_max (0.19 L min^−1^ and 2.7 ml kg^−1^ min^−1^) in POST following a 2‐week sprint interval training program that was similar to the training program utilized in this study. While this study did not observe significant improvements in $\dot{V}$O_2_max in PERI (0.09 L min^−1^ and 1.03 ml kg^−1^ min^−1^), previous investigations reported significant increases in $\dot{V}$O_2_max (~4 ml kg^−1^ min^−1^) in response to running‐based high‐intensity interval training (Koubaa et al., 2013) in 13‐year‐old males; however, this previous study did not quantify maturity status, which may explain the discrepancy in the results. Prior to puberty, the trainability of $\dot{V}$O_2_max in children appears to be blunted when compared to adults; however, there may still be slight improvements in aerobic exercise ability (Barker, Lloyd, Buchheit, Williams, & Oliver, 2014; Ford et al., 2011; Lloyd & Oliver, 2012). Previous research has demonstrated significant increases in $\dot{V}$O_2_max (2.50–5.51 ml kg^−1^ min^−1^) among PRE following running‐based sprint interval training programs (Baquet et al., 2010; Mucci et al., 2013). However, the results of this study were not supportive as no adaptations were observed in $\dot{V}$O_2_max in PRE (0.02 L min^−1^ and −0.01 ml kg^−1^ min^−1^) following the cycling‐based sprint interval training program. The discrepancies between this study findings and previous research with regards to PRE may be due to differences in training modality or duration. No previous investigation has assessed the efficacy of a cycling‐based sprint interval training program among PRE and PERI. Moreover, exercise intensity has been shown to be the most important variable in the trainability of aerobic performance among PRE, with intensities greater than 80% of maximum heart rate resulting in the greatest adaptations (Massicotte & Macnab, 1974). In this study, heart rate was not recorded during training; however, the intensity was considered “all‐out” which has been shown to improve maximal aerobic performance among POST (Barker, Day, et al., 2014), but has equivocal results among PRE (McManus, Cheng, Leung, Yung, & Macfarlane, 2005; Williams, Armstrong, & Powell, 2000). Future research should examine maturity‐related differences in the heart rate response during “all‐out” sprint interval training of different loads and duration. A review and meta‐analysis by Costigan et al. (2015) revealed that interval training program duration may be a significant moderator for changes in body composition, but did not investigate training program intensity or maturity as potential moderators.

This study observed significant increases in the systemic pulmonary fatigue thresholds, GET, VT, and RCP, within POST, but no changes in PRE or PERI. Furthermore, POST experienced greater adaptations in these fatigue thresholds than PRE or PERI. In terms of systemic pulmonary fatigue thresholds, Barker, Day, et al. (2014) observed a significant increase in GET, when expressed as an absolute $\dot{V}$O_2_ (0.09 L min^−1^) but not as a percent of $\dot{V}$O_2_peak, in POST; which was similar in magnitude to changes in this study (0.14 L min^−1^). Prior to puberty, lactate threshold (Rotstein et al., 1986), GET, and RCP (Mucci et al., 2013) have been shown to increase following interval training programs of at least 8 weeks, which is twice as long as the current training program. Furthermore, Faude et al. (2013) observed significant increases in individual anaerobic threshold among PERI after completing 16–20 sessions of high‐intensity interval training, high volume endurance training, or small‐sided soccer games, which is twice as many session as the current training program. While this study resulted in significant improvements in GET, VT, and RCP for only POST, it is possible that a longer training program, like those used in previous studies (Faude et al., 2013; Mucci et al., 2013; Rotstein et al., 1986), would result in changes for all maturity groups.

In terms of localized muscular fatigue thresholds, previous research has shown that improvements to neuromuscular activation can occur across all stages of maturation, which may explain the overall increase in NFT observed in this study (Viru et al., 1999). In terms of oxygenation, McNarry, Welsman, & Jones (2011) observed significant improvements in muscle deoxygenation responses and GET between trained and untrained girls at PRE, PERI, and POST, with similar differences between trained and untrained girls across all maturational groups, indicating no effect of maturity. The results of this study support this finding with a significant main effect in HHbBP, a fatigue threshold based on the deoxygenation of muscle, regardless of maturity group. While maturity may have an effect on the adaptations of systemic pulmonary fatigue thresholds, it does not appear that adaptations to localized muscular fatigue thresholds, such as NFT and HHbBP, are affected by maturity status. More research is needed to examine the role of maturity with longer duration training programs.

In terms of the anaerobic adaptations to sprint interval training, POST and PERI had significant increases in average PP and MP during the SIT sessions, while PRE did not experience any change in PP or MP. Barker, Day, et al. (2014) observed significant increases in PP, but not MP, following a similar sprint interval training in POST. Previous research has shown improvements in PP and MP within PRE following a 9‐week interval training program (Rotstein et al., 1986). However, Wright et al. (2016) observed decrements in repeated sprint ability within PRE and PERI, while POST had improvements in intermittent running performance and no decrements in repeated sprint ability following an 8‐week mixed‐mode interval training program. The possibility exists that all youth, regardless of maturity status, may improve muscular power output from interval training, but repeated anaerobic exercise may be affected by maturation. Inconsistencies in the literature regarding the anaerobic adaptations to interval training in youth are potentially due to a limited number of studies, training program variation, and differences in the assessment of anaerobic exercise performance (McNarry & Jones, 2014).

Previous research has purported that the trainability of aerobic and anaerobic fitness throughout maturation does not appear to be affected by a ‘maturational threshold’; however, anaerobic adaptations to training, such as strength and power, may be greater in more mature children (Barker, Lloyd, et al., 2014; Lloyd & Oliver, 2012). Additionally, there is a lack of research examining the role of sprint interval training during the natural ‘windows of opportunity’ that occur during maturation (Ford et al., 2011). While maximal aerobic performance appears to be trainable throughout maturation (Barker, Lloyd, et al., 2014; Lloyd & Oliver, 2012), it has been well‐established that children have diminished anaerobic capabilities prior to puberty (Armstrong, Welsman, & Kirby, 1997; Beneke et al., 2007). This diminished anaerobic capacity in PRE may be due to several factors including lower glycogen storage at rest and utilization during exercise (Eriksson & Saltin, 1974), and lower activities of anaerobic enzymes phosphofructokinase and lactate dehydrogenase (Eriksson, Gollnick, & Saltin, 1973). While this study did not investigate these factors, evidence of this diminished anaerobic capacity was observed in the current training program, as PERI and POST completed significantly more work during training than PRE, even when controlling for body mass. It is possible that the maturity‐related differences in the adaptations to sprint interval training are due to the limited anaerobic capacity prior to puberty, which would reduce the training stimulus in PRE, rather than a lack of trainability among children who have not reached puberty. However, it is important to note that PERI experience no changes in maximal aerobic performance or fatigue thresholds despite completing similar relative total work during training to POST. Furthermore, the absolute change in VO_2_peak between PRE (0.03 ± 0.09 L min^−1^) and PERI (0.04 ± 0.20 L min^−1^) was statistically similar despite PERI completing a significantly greater relative total work during training.

In conclusion, the sprint interval training program utilized in this study may be an effective means to improve maximal aerobic performance and fatigue thresholds in POST, while also improving repeated anaerobic performance in PERI and POST. The maturity‐related differences in the adaptations to high‐intensity interval training may be due to the design of the training program, the trainability of the measures within each maturational group, or the differences in performance during the training program. While the current sprint interval training program may be a time‐efficient way to improve aerobic and anaerobic exercise performance during adolescence, more research is needed to determine the optimal high‐intensity interval training program design to elicit the greatest adaptations across all maturational groups.
